# Pediatric high users of Canadian hospitals and emergency departments

**DOI:** 10.1371/journal.pone.0251330

**Published:** 2021-05-06

**Authors:** Ryan Tiller, Kevin Chan, John C. Knight, Roger Chafe

**Affiliations:** 1 Faculty of Medicine, Memorial University of Newfoundland, St. John’s, Newfoundland and Labrador, Canada; 2 Department of Pediatrics, University of Toronto, Toronto, Ontario, Canada; 3 Trillium Health Partners, Mississauga, Ontario, Canada; 4 Newfoundland and Labrador Centre for Health Information, St. John’s, Newfoundland and Labrador, Canada; 5 Janeway Pediatric Research Unit, St. John’s, Newfoundland and Labrador, Canada; University of Mississippi Medical Center, UNITED STATES

## Abstract

**Introduction:**

Few studies have examined the most frequent pediatric users of hospital services. Our objective was to determine the clinical diagnoses, demographic characteristics, and medical severity of high-use pediatric patients in Canada.

**Methods:**

We conducted a retrospective analysis of patients <18 years of age who either were admitted to hospital or visited an emergency department (ED) using the Canadian Institute for Health Information’s (CIHI) *Dynamic Cohort of Complex*, *High System Users*. The analysis of hospital admission data excluded Quebec and Manitoba. ED data was only available for Alberta and Ontario.

**Results:**

121 104 patients were identified as the most frequent hospital users and 459 998 patients as the most frequent ED users. High users were more likely to reside in a rural community, to be in a lower income quintile, and face more deprivation. The most frequent conditions for hospitalization for high use patients were disorders related to length of prematurity and fetal growth, respiratory and cardiovascular disorders specific to the perinatal period, and haemorrhagic and haematological disorders of fetus and newborn. For the most frequent ED users, the most common clinical diagnoses were acute upper respiratory infections, injuries to the head, and diseases of the middle ear and mastoid.

**Conclusion:**

Pediatric high users by frequency of hospital and ED services are a distinct population. Better understanding their characteristics will allow for more appropriate planning of children’s health services and help identify areas for effective preventive or quality improvement initiatives.

## Introduction

Frequent pediatric health care service users have different health issues than adult high users. Yet previous work on high use pediatric patients is limited and mostly focused on frequent emergency department (ED) users. The *Canadian Institute for Health Information* (CIHI) recently reported that frequent ED users under 18 years of age, defined as people having more than 4 ED visits in a year, accounted for 21.2% of all ED visits for that age group [1]. Seguin et al. found at a pediatric ED in Montreal, Canada, that the most frequent users were younger and had lower socioeconomic status; with the most frequent diagnoses being ear, nose and sinus disorders; infectious respiratory diseases; and asthma [2]. Supat et al. found that the majority of pediatric frequent ED users in California did not seek care in a pediatric-specific ED, with upper respiratory infections (13.2%), asthma (4.7%), and abdominal pain (4.5%) being the most frequent diagnosis [3]. None of the previous studies have looked at the high user pediatric population across multiple provinces nor examined the most frequently hospitalized pediatric patients.

In this study, we analyze data from the *CIHI*’s *Dynamic Cohort of Complex*, *High System Users* to determine the most common medical conditions pediatric high users present with [4]. We then compared these pediatric high users of hospitals and EDs to controls to better understand their unique clinical and socio-demographic characteristics.

## Methods

### Data

The *Dynamic Cohort of Complex*, *High System Users* includes information on patients who had at least one acute care hospitalization or ED visit during the fiscal years 2011/12 to 2014/15. We only accessed the data files for children (defined as <18 years old), which are separate from the data sets for adults. This data captures patient encounters at both pediatric-specific and general hospitals.

For hospitalizations, data is from the Discharge Abstract Database (DAD), which is reported to CIHI by all provinces and territories except Quebec. We analyzed data from eight provinces and the three territories. Complete and consistent data for Manitoba was not included in the dynamic cohort data to which we had access, so we excluded it from our analysis. The hospitalization data captures discharges from hospitals and therefore excludes short inpatient visits. For ED visits, data is from the National Ambulatory Care Reporting System (NACRS), which was only available for the provinces of Ontario and Alberta during the study period.

The data used in this study are from the *CIHI’s Dynamic Cohort of Complex*, *High System Users*. Applications to access the data set need to be made to CIHI (see https://cihr-irsc.gc.ca/e/50129.html). Additional inquiries can be made to CIHI’s Corporate Data Request Program at cdrp@cihi.ca. The authors had no special access privileges and other researchers will be able to access the data in the same manner as the authors.

### High user definition

The Dynamic Cohort identifies patients as a most frequent user for either hospital admissions or ED visits. To be categorized as a most frequent user, patients need to be in the top 10% of pediatric patients in terms of frequency of either hospital admissions or ED visits respectively in any one of the four years covered by the cohort, e.g., a ED frequent user were in the top 10% of ED users in at least one of the four years examined.

In conducting our analysis, we identified patients who would not normally be considered a frequent user. For example, the cohort identified 591 (0.13%) patients with only one or two ED visits for a four-year period as a most frequent user. To remove these inappropriate cases, we restricted our high user group to patients who both met the Dynamic Cohort’s definition of a frequent ED user and had at least 3 ED visits over the four-year period [5].

The cohort also included those whose only hospitalization was their birth. Given that studies of adult high use of care often exclude hospitalizations involving an obstetrics delivery, we excluded birth hospitalizations with a most-responsible diagnosis of “live born infant”, i.e., a healthy live birth with no other health issues, for both the high user and control groups. We did not exclude birth hospitalizations with a most-responsible diagnosis other than “live-born infant”, i.e., more complicated live births, as we expected these patients would be more frequent users of pediatric care. By doing this, we removed 177 969 (47.5%) patients who had no other hospitalization except their healthy birth from both the high user and control groups, as they no longer met the definition of a hospitalized patient.

The control groups for the Dynamic Cohort were a randomly selected sample of pediatric patients who were not high user in any of the four years in the study period. The controls for the hospitalization group had at least one hospitalization over the four-year period. The controls for the ED group had at least one ED visit over the four-year period. Controls were provided by CIHI as part of the cohort data [4].

Variables reported for these patients included sex, age, postal code, hospitalizations, ED visits, hospital length of stay (LOS), comorbidity score [6], total hospital cost, andmost-responsible diagnosis / main complaint. A resource intensity weight (RIW), which is a relative value that describes expected resource use of a patient, was also reported [7].Where patients had multiple hospitalizations in the study period, the highest co-morbidity level and RIW values were used in the analysis. All analyses were conducted at the patient level.

### Statistical analysis

We conducted separate analyses for hospital and ED high users. For both analyses, we pooled the available data from the provinces and territories together. We analyzed the high user data and control data over the four-year period. We conducted sensitivity analysis that separated data by year and found similar results to those reported here, particularly around the most common diagnosis.

The patient characteristics used in the analysis were those for the patient’s earliest hospitalization or ED visit identified in the cohort. Income quintiles were determined based on postal code. Census metropolitan area (CMA) and census agglomeration (CA) were also determined using postal codes [8]. The level of social inequality was assessed using the INSPQ Deprivation Index, which captures a combination of material and social deprivation measures [9, 10]. The lower the Deprivation Index the greater level of deprivation a person faces. In order to analyze the main/most-responsible diagnosis, we categorized the medical conditions using ICD-10-CA blocks [11].

Frequencies were calculated for categorical variables. Because count variables (e.g., ED visits and hospitalizations) and continuous variables (e.g., length of stay, RIW, cost) were skewed, medians and interquartile ranges were calculated for these variables. Chi-squared tests were used to examine differences in frequencies for categorical variables and Mann-Whitney U tests were used for count and continuous variables. Statistical analyses were conducted using SPSS V. 21.0 [12].

Multivariate binary logistic regression was used to determine factors predicting being a high hospital user and a high ED user (two separate models) using the factors in Table 1 and the top ten diagnoses for high users in Tables 2 and 3. Deprivation quintiles were excluded from the regression analysis as they were only available in CMAs. In addition, RIW and hospital cost were also excluded from the hospital regression analysis due to high collinearity with other variables in the model (correlation coefficient (r^2^) > 0.6). All other factors were found to be significant predictors of high use in unadjusted models (p <0.05) and thus were included in forced multivariate models. We carried out regression analyses using SAS Enterprise Guide version 7.1 (SAS Institute Inc.).

**Table 1:** Sociodemographic and morbidity characteristics of hospital and ED high users.

Characteristic	Hospitalizations			ED Visits		
	High Users	Controls	p-value	High Users	Controls	p-value
	(n = 121,104)	(n = 78,404)		(n = 459,998)	(n = 341,631)	
% Male	53.2	53.0	0.812	52.5	53.2	<0.001
Age Group			<0.001			<0.001
Newborn	48.6	58.2		0.0	0.0	
0–28 Days	0.7	0.3		4.3	2.2	
1–11 Months	4.7	1.3		16.3	9.7	
1–4 years	12.2	10.3		29.2	27.0	
5–9 years	8.6	11.0		16.7	24.9	
10–14 years	11.7	9.9		18.4	24.3	
> 14 years	13.5	8.9		15.2	11.9	
% Urban	78.0	84.3	<0.001	71.0	85.2	<0.001
National Income Quintile			<0.001			<0.001
Q1 (Lowest)	21.0	18.0		14.7	11.6	
Q2	19.5	17.7		17.9	14.3	
Q3	19.6	20.2		20.1	18.7	
Q4	19.9	21.6		22.3	23.9	
Q5 (Highest)	19.9	22.5		23.5	30.3	
Number of events per person [median (IQR)]	Hospitalizations			ED Visits		
	2.0 (2)	1.0 (0.0)	<0.001	5.0 (4.0)	1.0 (1.0)	
Highest Hospital Co-morbidity Level	56.3	86.3	<0.001			
0 (No co-morbidity)						
1	10.1	5.1				
2	11.5	4.7				
3	11.2	3.1				
4 (High co-morbidity)	10.9	0.8				
Hospital Length of Stay (days) [median (IQR)]	8.0 (16.0)	2.0 (2.0)	<0.001			
Highest Hospital RIW [median (IQR)]	1.04 (2.23)	0.37 (0.36)	<0.001			
Cost of Hospital Stay (CAD $) [median (IQR)]	10,901 (23,499)	2303 (2128)	<0.001			
Deprivation Quintiles (CMA Residents only)	(n = 60,936)	(n = 44,340)		(n = 197,363)	(n = 196,319)	
Overall Deprivation			<0.001			<0.001
Q1 (Least Deprived)	16.7	16.4		16.7	20.7	
Q2	18.7	19.2		19.1	20.6	
Q3	19.5	21.1		18.1	18.7	
Q4	19.4	19.6		18.4	17.4	
Q5(Most Deprived)	25.7	23.7		27.6	22.6	
Material Deprivation			0.009			<0.001
Q1 (Least Deprived)	16.0	16.4		14.6	18.1	
Q2	18.4	18.3		18.9	19.4	
Q3	20.0	20.1		20.2	20.0	
Q4	20.7	21.2		21.1	20.3	
Q5(Most Deprived)	24.9	24.0		25.2	22.1	
Social Deprivation			<0.001			<0.001
Q1 (Least Deprived)	18.5	19.3		18.2	21.6	
Q2	8.0	18.4		17.9	19.4	
Q3	19.8	19.6		19.9	20.2	
Q4	21.6	21.6		21.7	20.5	
Q5 (Most Deprived)	22.1	21.1		22.2	18.3	

**Table 2:** Ten most common clinical diagnoses of high hospital users.

ICD-10-CA Block	High User	Control
	n = 121,104 Rank (%*)	n = 78,404
Disorders related to length of gestation and fetal growth	1 (23.7)**	1 (20.0)
Respiratory and cardiovascular disorders specific to the perinatal period	2 (11.7)	2 (14.0)
Haemorrhagic and haematological disorders of fetus and newborn	3 (9.8)**	3 (5.9)
Other acute lower respiratory infections	4 (8.3)**	20 (1.2)
Influenza and pneumonia	5 (7.0)**	7 (2.4)
General symptoms and signs	6 (6.8)**	15 (1.5)
Persons encountering health services for specific procedures and health care	7 (6.3)**	29 (0.9)
Intestinal infectious diseases	8 (5.0)**	22 (1.1)
Mood [affective] disorders	9 (4.7)**	9 (2.3)
Acute upper respiratory infections	10 (4.2)**	18 (1.4)

* Indicates percentage of people who have at least one hospitalization/ED visit with that condition.

** Indicates percentage significantly different from control at α = 0.001.

**Table 3:** Ten most common clinical diagnoses of high ED users.

ICD-10-CA Block	High User	Control
	n = 459,998 Rank (%*)	n = 341,631
Acute upper respiratory infections	1 (48.4)**	1 (18.5)
Injuries to the head	2 (25.9)**	2 (17.8)
Diseases of middle ear and mastoid	3 (22.3)**	7 (7.3)
General symptoms and signs	4 (21.6)**	5 (7.8)
Symptoms and signs involving the digestive system and abdomen	5 (20.0)**	6 (7.5)
Intestinal infectious diseases	6 (16.9)**	9 (6.0)
Injuries to the wrist and hand	7 (16.0)**	3 (9.5)
Other viral diseases	8 (16.0)**	10 (5.1)
Symptoms and signs involving the circulatory and respiratory systems	9 (14.2)**	12 (4.4)
Injuries to the ankle and foot	10 (13.2)**	4 (7.9)

* Indicates percentage of people who have at least one hospitalization/ED visit with that condition.

** Indicates percentage significantly different from control at α = 0.001.

Ethical approval for the study was granted by the University of Calgary’s Conjoint Health Research Ethics Board [Ethics ID: REB152493]. The data in *CIHI*’s *Dynamic Cohort of Complex*, *High System Users* is fully anonymized. There was no requirement to get informed consent from patients prior to analyzing the cohort data.

## Results

### Hospital analysis

There were 199 508 patients in the cohort we analyzed: 121 104 (60.7%) high users and 78 404 (39.3%) controls (*Table 1*). There was no significant difference in gender between the high user group and controls (53.2% vs 53.0% male, *p* = 0.812). High users were more likely to reside in a rural community (22.0% vs 15.7%, *p* < 0.001), to be in a lower income quintile (p <0.001), and to have slightly higher levels of deprivation (p <0.001).

The high users accounted for 397 480 (82.5%) hospitalizations, with a median of 2 hospitalizations per person (interquartile range [IQR] 2) over the four-year period (Range 1–63). The cohort controls accounted for 84 195 (17.5%) hospitalizations with a median of 1 hospitalization per person (IQR 0.0; Table 1) (Range 1–4). High users were more likely to have a higher comorbidity level (p<0.001), higher median length of stay (8 days [IQR 5] vs 2 days [IQR 2], *p* < .001), higher RIW (1.04 [IQR 2.23] vs 0.378 [IQR 0.36], *p* < .001), and higher hospital cost compared to controls (CAD $10,901 [IQR 23,499] vs CAD $ 2,303 [IQR 2,128] *p* < .001). Because of missing National Income Quintile data, less than 2%, the percentages in Table 1 do not add to 100%.

*Table 2* ranks the 10 most frequent most-responsible diagnoses for high users and controls. The most common medical conditions for the high hospitalization group were disorders related to prematurity and fetal growth (23.7%), respiratory and cardiovascular disorders specific to the newborn (11.7%), haemorrhagic and haematological disorders of fetus and newborn (9.8%), and other acute lower respiratory infections (8.3%). All 10 conditions were more common in the high users than in the controls. The top three conditions and the mood [affective] disorders (9^th^) were ranked the same in both groups while the remaining conditions ranked lower in controls than in the high users.

### ED analysis

The cohort included 801 629 pediatric patients who were seen in an ED: 459 998 (57.4%) high users and 341 631 (42.6%) controls (*Table 1*). The high users accounted for 3 036 992 (83.0%) ED visits with a median of 5 visits per person (interquartile range [IQR] 4) (Range 3–283). The controls accounted for 619 885 (17.0%) visits with a median of 1 visit per person (IQR 1) (Range 1–24).

High ED users were more likely to be female (47.5% vs 46.8%, *p* < .001) or to be from a rural community as compared to controls (29.0% vs 14.8%, *p* < .001). High ED users were more likely to be in a lower income quintile (<0.001), and to have greater deprivation levels (<0.001).

*Table 3* ranks the 10 most frequent main complaints (i.e., diagnoses) for ED high users and controls. The most common medical conditions for the high ED user group were acute upper respiratory infections (48.4%), injuries to the head (25.9%), and diseases of middle ear and mastoid (22.3%). All 10 conditions were more common in the high users than in the controls. The top two conditions, acute upper respiratory infections and injuries to the head, ranked the same in both groups while the remaining conditions ranked lower in controls than in the high users, except for injuries to the wrist and hand and injuries to the ankle and foot, which actually ranked higher in controls.

### Multivariate logistic regression analysis

#### Hospital analysis

Table 4 presents the results of a binary multivariate logistic regression showing odds ratios for factors predicting being in the high hospital user group. A female was 5.4% less likely to be in the high user groups than a male. Relative to newborns, individuals in other age groups were more likely to be high users ranging from 41.7% more likely in the 0–28 days age group to 5.16 times more likely in the 1–11 months age group. Individuals from urban areas were 35% less likely to be high users than those from rural areas. Relative to the lowest income quintile, individuals in other income quintiles were less likely to be high users with a trend towards higher income quintiles being less likely to be high users. Relative to no comorbidity, individuals with co-morbidities were more likely to be high users with a trend towards individuals with higher co-morbidity levels being more likely to be high users. An individual was about 30% more likely to be a high user for each additional day of hospital stay. Individuals having any of the top ten diagnoses for the high user group were more likely to be in the high user group, ranging from 15% more likely for individuals with disorders related to length of gestation and fetal growth to over 6 times as likely for those with other acute lower respiratory infection.

**Table 4:** Predictors of being a high hospital user.

**Parameter**	**Odds Ratio (95% confidence interval)**
**Sex**	
Male (Ref.)	**1.000**
Female	0.946 (0.924–0.968)
**Age Group (years)**	
Newborn (Ref.)	**1.000**
0–28 days	1.417 (1.179–1.704)
1–11 months	5.164 (4.764–5.599)
1–4 years	2.727 (2.609–2.852)
5–9 years	2.217 (2.119–2.319)
10–14 Years	2.420 (2.313–2.532)
> 14 years	3.130 (2.991–3.275)
**Rural-Urban Status**	
Rural (Ref.)	**1.000**
Urban	0.640 (0.621–0.660)
**National Income quintile**	
Q1 (Lowest) (Ref.)	**1.000**
Q2	0.988 (0.952–1.025)
Q3	0.887 (0.855–0.920)
Q4	0.835 (0.805–0.866)
Q5 (Highest)	0.795 (0.766–0.825)
**Hospital Variables**	
Co-morbidity Level	
0 (No co-morbidity) (Ref.)	**1.000**
1	1.576 (1.504–1.651)
2	1.726 (1.645–1.811)
3	1.637 (1.545–1.736)
4 (Highest co-morbidity)	2.148 (1.943–2.374)
Hospital Length of Stay	1.305 (1.300–1.311)
**Top Ten Diagnoses**	
1) Disorders related to length of gestation and fetal growth	1.151 (1.108–1.196)
2) Respiratory and cardiovascular disorders in the perinatal period	1.593 (1.528–1.660)
3) Hemorrhagic and hematological disorders of fetus/newborn	5.117 (4.905–5.337)
4) Other acute lower respiratory infections	6.154 (5.710–6.633)
5) Influenza and pneumonia	1.723 (1.619–1.834)
6) General symptoms and signs	3.398 (3.165–6.647)
7) Persons encountering health services for procedures/healthcare	4.858 (4.441–5.315)
8) Intestinal infectious diseases	1.907 (1.787–2.034)
9) Mood [affective] disorders	1.253 (1.146–1.371)
10) Acute upper respiratory infections	2.660 (2.464–2.872)

### ED analysis

Table 5 presents the results of a binary logistic regression showing odds ratios for factors predicting being in the high ED user group. Gender was not a significant predictor of high ED use. Relative to newborns, individuals in the 5–10 year age group were 43% less likely to be a high ED user while other groups were not significantly different from newborns. Individuals from urban areas were about 53% less likely to be high users than those from rural areas. Relative to the lowest income quintile, individuals in other income quintiles were less likely to be high users with a trend towards higher income quintiles being less likely to be high users. Individuals having any of the top ten diagnoses for the high user group were more likely to be in the high user group, ranging from almost 97% more likely for individuals with acute lower respiratory infection to over 3.7 times as likely for those with symptoms and signs involving the digestive system or abdomen.

**Table 5:** Predictors of high being a high ED user.

Parameter	Odds Ratio (95% confidence interval)
**Sex**	
Male (Ref.)	**1.000**
Female	0.992 (0.982–1.003)
**Age Group (years)**	
Newborn (Ref.)	**1.000**
0–28 days	1.349 (0.907–2.007)
1-11 months	0.812 (0.546–1.207)
1–4 years	0.679 (0.469–1.035)
5-9 years	0.570 (0.383–0.846)
10-14 ears	0.740 (0.498–1.110)
> 14 years	1.382 (0.930–2.054)
**Rural-Urban Status**	
Rural (Ref.)	**1.000**
Urban	0.479 (0.472–0.486
**National Income quintile**	
Q1 (Lowest)	**1.000**
Q2	0.949 (0.930–0.968)
Q3	0.891 (0.874–0.908)
Q4	0.865 (0.849–0.881)
Q5 (Highest) (Ref.)	0.826 (0.811–0.841)
**Top Ten Diagnoses**	
1) Acute upper respiratory infections	3.784 (3.738–3.831)
2) Injuries to the head	1.968 (1.959–2.012)
3) Diseases of middle ear and mastoid	3.187 (3.132–3.243)
4) General symptoms and signs	3.357 (3.301–3.413)
5) Symptoms and signs involving the digestive system/abdomen	3.736 (3.673–3.800)
6) Intestinal infectious diseases	2.892 (2.837–2.948)
7) Injuries to the wrist and hand	2.564 (2.521–2.607)
8) Other viral diseases	3.046 (2.985–3.107)
9)Symptoms and signs involving circulatory/respiratory systems	3.423 (3.352–3.496)
10) Injuries to the ankle and foot	2.296 (2.255–2.338)

## Discussion

This study is one of the first to examine pediatric hospital and ED high use patients at a national level. We found that pediatric high users of both hospitals and EDs were more likely to be in a lower income quintile and to face more deprivation. The most frequent users of hospitals were also sicker compared to controls, with higher levels of comorbidity, longer lengths of stay, and higher costs per hospitalization. The most frequently hospitalized patients do not just come to hospital more often, they are sicker and require more care. The most common medical conditions for the high hospitalization group were neonatal related disorders. The most common medical conditions for the high ED user group were acute upper respiratory infections, head injuries, and diseases of the middle ear and mastoid. Almost half of all individuals in the high user group had at least one ED visit for an acute upper respiratory infection. Supporting these findings, Supat et al. (2018) found upper respiratory infections to be the most common diagnosis in frequent pediatric ED users in California [3].

Pediatric high users of both the hospital and ED tended to be from more socio-economically disadvantaged and rural areas when compared to the cohort’s controls. This relationship between socioeconomic status and hospital usage has been shown in other studies [13, 14]. Wood et al. assessed the relationship between welfare status, health insurance status and health care use among children with asthma [15]. They found that children of families in the United States who had applied for social assistance and were uninsured had more asthma acute care visits [15]. Rural areas are known to have less access to health care services, including pediatric specialists and pediatric-specific hospitals [16]. One potential focus for future policy work would be to look at models of care delivery and increased social supports for disadvantaged rural families of high use patients.

Previous studies have shown that younger patients, specifically neonates, had higher rates of admission [17]. It is also known that hospitalizations related to prematurity are expensive and can require long hospital stays. While we excluded healthy live births, we found conditions related to premature births and neonates are leading causes of hospitalizations. Given that the data is from both pediatric focused and general hospitals, the frequency of neonate patients and their complex health issues highlight a key patient population on which to focus quality improvement initiatives. We found a relationship between the degree of severity of a patient condition (i.e., length of stay, RIW, and comorbidity score) and frequency of hospital usage that has been identified in other studies [6]. Programs targeting this subset of the population may also have the potential to reduce health care spending.

Several conditions did not make the list of the most frequent diagnosis of high users. Both Seguin et al. and Supat et al. found that asthma was one of the most common diagnosis given in ED [2, 3]. In our cohort, asthma was the 15th most common diagnosis amongst ED high users representing 2.2% of cases. We focused only on the most responsible diagnosis. It is possible that this missed some cases where asthma is listed as a comorbidity. Similarly, none of the most frequent diagnosis for ED users and only one for high hospitalization users are related to mental health issues. We have recently found a significant increase of self-poisoning hospitalizations over a five year period at one pediatric hospital [18]. CIHI reported hospitalization data from the Ontario Mental Health Reporting System which showed several mental health conditions in the most frequent diagnoses within the 5–17 age group [19]. Given the increased rates of mental health issues in the adolescent population, more focus on mental health diagnosis, and better data capture, mental health issues will likely represent greater percentages of pediatric high user hospitalizations and ED visits in the future.

We made several choices in how we analyzed the data. We analyzed the cohort for the entire 4-year period instead of analyzing each year separately. Yearly changes in severity and prevalence of illnesses and infections could potentially skew the results. In ordered to analyze the main/most-responsible diagnoses, we categorized the medical conditions using ICD-10-CA blocks. This allowed for comparison across diagnoses, but precludes us focusing on more specific diagnoses below this level of categorization. Wick et al. compared three definitions of high-use hospital patients: in terms of number of inpatient episodes (which is the approach we used), cumulative length of stay, and cumulative cost [20]. They found that high-use patients based on a high number of inpatient episodes were more likely to be admitted for acute conditions, but when based on length of stay hospitalizations were more likely to be admitted for mental health-related conditions.

## Limitations

The study had several limitations. While CIHI’s Dynamic Cohort provides a unique opportunity to examine pediatric high users on a national level, the period covered by the cohort ended five years ago, so that it may not be representative of current conditions. While the cohort identified most frequent users, issues with the some of patients identified in the cohort led us to put additional restrictions our definition of high users. The number of high users in the cohort appear to be disproportionately large when compared with the number of controls. Given the sample size and the strict definition used in selecting controls and high users, it is not clear how this relative mismatch may have impacted our findings. While the cohort indicated the specific year for which patients were a high user, controls were not identified by year, restricting our ability to do year by year analysis. While the study describes pediatric high users of hospitals representing about 74% of the Canadian population, data from Quebec and Manitoba were not included [21]. Our analysis of pediatric ED users was limited to data from Alberta and Ontario, which represents only approximately 50% of the Canadian population. We did not conduct any cross provincial comparisons to determine regional variations, as this was outside the scope of our original research focus. We also did not use any corrections for multiple testing, but the observed differences we found were so highly significant that we do not feel that its inclusion would have meaningfully impacted our results.

## Conclusion

Like high users of adult health care services, pediatric hospital and ED high users represent a critical population for our health care system to understand. The present study described the clinical diagnoses, socio-demographic characteristics, and degree of severity for pediatric high use patients in Canadian hospitals and EDs. Our results are useful for planning quality improvement initiatives aimed at this subset of the population. Achieving these goals will likely require even greater understanding of this patient population. Further research should focus on capturing a more comprehensive picture of who these high user patients are, how they change over time and how we can support them better.

## References

[1] CIHI. NACRS Emergency Department Visits and Length of Stay by Province/Territory, 2018–2019. https://www.cihi.ca/en/nacrs-emergency-department-visits-and-length-of-stay-2018-2019-xlxs. Published 2019. Accessed September 16, 2020.

[2] Seguin J, Osmanlliu E, Zhang X, et al. Frequent users of the pediatric emergency department. *Can J Emerg Med*. 2018;20(3):401–408. 10.1017/cem.2017.15 28382879

[3] Supat B, Brennan JJ, Vilke GM, Ishimine P, Hsia RY, Castillo EM. Characterizing pediatric high frequency users of California emergency departments. *Am J Emerg Med*. 2019. 10.1016/j.ajem.2018.12.015 30651182 PMC6561830

[4] CIHI. Dynamic Cohort of Complex, High System Users– 2011–2015—CIHR. https://cihr-irsc.gc.ca/e/50129.html. Accessed April 19, 2020.

[5] Giannouchos T-V., Washburn DJ, Gary JC, Foster MJ. Frequent emergency department use in the paediatric population: A systematic literature review. *J Eval Clin Pract*. 3 2020:jep.13382. 10.1111/jep.13382 32141125

[6] CIHI. *DAD Data Elements 2015–2016*. https://www.cihi.ca/sites/default/files/document/dad_data_elements_2015_2016_en.pdf. Accessed April 21, 2020.

[7] Canadian Institute for Health Information. *The Cost of Hospital Stays: The Cost of Hospital Stays: Why Costs Vary.*; 2008. https://secure.cihi.ca/free_products/2008hospcosts_report_e.pdf. Accessed April 21, 2020.

[8] Statistics Canada. Dictionary, Census of Population, 2016—Census metropolitan area (CMA) and census agglomeration (CA). https://www12.statcan.gc.ca/census-recensement/2016/ref/dict/geo009-eng.cfm. Published 2016. Accessed September 16, 2020.

[9] CIHI. *Data and Analysis Methodology.*; 2008. www.cihi.ca. Accessed April 21, 2020.

[10] Herring J. *Summary Measures of Socioeconomic Inequalities in Health.*; 2013. www.publichealthontario.ca. Accessed September 16, 2020.

[11] CIHI. *Canadian Coding Standards for Version 2018 ICD-10-CA and CCI.*; 2018. www.cihi.ca. Accessed April 19, 2020.

[12] SPSS Statistics—Overview | IBM. https://www.ibm.com/products/spss-statistics?p1=Search&p4=p50436828401&p5=b&cm_mmc=Search_Google-_-1S_1S-_-WW_NA-_-%2Bspss %2Bstats_b&cm_mmca7=71700000060952240&cm_mmca8=aud-309367918490:kwd-305473635268&cm_mmca9=CjwKCAjw8J32BRBCEiwApQEKgQD_ImBKuLKSONT2Q_fuAqTQDufOyFsV44eqUttIK72Htf5EX9RwKRoCKJEQAvD_BwE&cm_mmca10=407036424940&cm_mmca11=b&gclid=CjwKCAjw8J32BRBCEiwApQEKgQD_ImBKuLKSONT2Q_fuAqTQDufOyFsV44eqUttIK72Htf5EX9RwKRoCKJEQAvD_BwE&gclsrc=aw.ds. Accessed May 22, 2020.

[13] Berry JG, Hall DE, Kuo DZ, et al. Hospital Utilization and Characteristics of Patients Experiencing Recurrent Readmissions Within Children’s Hospitals. *JAMA—J Am Med Assoc*. 2011;305(7):682–690. 10.1001/jama.2011.122 21325184 PMC3118568

[14] Wodchis WP, Austin PC, Mbchb DAH, Wodchis W. A 3-year study of high-cost users of health care. *CMAJ*. 2016;(3):188. 10.1503/cmaj.150064 26755672 PMC4754179

[15] Wood PR, Smith LA, Romero D, Bradshaw P, Wise PH, Chavkin W. Relationships between welfare status, health insurance status, and health and medical care among children with asthma. *Am J Public Health*. 2002;92(9):1446–1452. 10.2105/ajph.92.9.1446 12197971 PMC1447256

[16] Sibley LM, Weiner JP. *An Evaluation of Access to Health Care Services along the Rural-Urban Continuum in Canada*.; 2011. 10.1186/1472-6963-11-20 21281470 PMC3045284

[17] Perry AM, Caviness AC, Allen JY. Characteristics and diagnoses of neonates who revisit a pediatric emergency center. *Pediatr Emerg Care*. 2013;29(1):58–62. 10.1097/PEC.0b013e31827b540e 23283265

[18] Chafe R, Aslanova R, Hamud O, Gregory P, Newhook LA. Hospitalizations due to self-poisoning at a Canadian paediatric hospital. *Paediatr Child Health*. 2017;23(2):101–105. 10.1093/pch/pxx149 29686493 PMC5905369

[19] CIHI. Inpatient Hospitalization, Surgery and Newborn Statistics. 2020. https://www.cihi.ca/en/hospital-stays-in-canada. Accessed September 16, 2020.

[20] Wick J, Hemmelgarn B, Manns B, et al. Comparison of Methods to Define High Use of Inpatient Services Using Population-Based Data. *J Hosp Med*. 2017;12(8):596–602. 10.12788/jhm.2778 28786424

[21] Statistics Canada. Population—Canada at a Glance, 2018. https://www150.statcan.gc.ca/n1/pub/12-581-x/2018000/pop-eng.htm. Accessed April 22, 2020.

